# Improving the Resolution and Accuracy of Groundwater Level Anomalies Using the Machine Learning-Based Fusion Model in the North China Plain

**DOI:** 10.3390/s21010046

**Published:** 2020-12-24

**Authors:** Gangqiang Zhang, Wei Zheng, Wenjie Yin, Weiwei Lei

**Affiliations:** 1 School of Surveying and Landing Information Engineering, Henan Polytechnic University, Jiaozuo 454000, China; 211804020040@home.hpu.edu.cn (G.Z.); wwlei@hpu.edu.cn (W.L.); 2 Qian Xuesen Laboratory of Space Technology, China Academy of Space Technology, Beijing 100094, China; yinwenjie@qxslab.cn; 3 School of Geomatics, Liaoning Technical University, Fuxin 123000, China; 4 School of Aerospace Science and Technology, Xidian University, Xi’an 710126, China; 5 School of Instrument Science and Engineering, Southeast University, Nanjing 210096, China

**Keywords:** machine learning-based fusion model, GRACE, gradient boosting decision tree, groundwater level anomalies, statistical downscaling, North China Plain

## Abstract

The launch of GRACE satellites has provided a new avenue for studying the terrestrial water storage anomalies (TWSA) with unprecedented accuracy. However, the coarse spatial resolution greatly limits its application in hydrology researches on local scales. To overcome this limitation, this study develops a machine learning-based fusion model to obtain high-resolution (0.25°) groundwater level anomalies (GWLA) by integrating GRACE observations in the North China Plain. Specifically, the fusion model consists of three modules, namely the downscaling module, the data fusion module, and the prediction module, respectively. In terms of the downscaling module, the GRACE-Noah model outperforms traditional data-driven models (multiple linear regression and gradient boosting decision tree (GBDT)) with the correlation coefficient (CC) values from 0.24 to 0.78. With respect to the data fusion module, the groundwater level from 12 monitoring wells is incorporated with climate variables (precipitation, runoff, and evapotranspiration) using the GBDT algorithm, achieving satisfactory performance (mean values: CC: 0.97, RMSE: 1.10 m, and MAE: 0.87 m). By merging the downscaled TWSA and fused groundwater level based on the GBDT algorithm, the prediction module can predict the water level in specified pixels. The predicted groundwater level is validated against 6 in-situ groundwater level data sets in the study area. Compare to the downscaling module, there is a significant improvement in terms of CC metrics, on average, from 0.43 to 0.71. This study provides a feasible and accurate fusion model for downscaling GRACE observations and predicting groundwater level with improved accuracy.

## 1. Introduction

As a significant supply source of freshwater resources, groundwater plays a crucial role in social production and human life [1,2]. Globally, it provides drinking water for approximately two billion people [3] and irrigation for roughly 40% of areas equipped for irrigation [4]. Due to extreme climate episodes and anthropogenic actives (e.g., drought and overuse of irrigation water), groundwater resources are seriously over-exploited in some typical regions [5,6], leading to a series of environmental issues, such as land subsidence and seawater intrusion [7,8]. Therefore, understanding the dynamics changes in groundwater is necessary for the effective utilization and sustainable management of water resources [2,9].

The traditional method to monitor groundwater levels is based on ground-based measurements [10,11]. However, it is not applicable to large-scale and remote regions restricted by national policies, limited stations, and instrument accuracy. The Gravity Recovery and Climate Experiment (GRACE) satellites, successfully launched in March 2002 [12], provides a kind of new method for monitoring the global time-variable gravity field with unprecedented accuracy [13]. Additionally, it can provide continuous terrestrial water storage anomalies (TWSA) and cover most parts of the world, which is especially beneficial for areas lacking ground-based measurements. By integrating auxiliary information from hydrological models, groundwater storage anomalies (GWSA) can be further isolated from GRACE observations. Previous studies have demonstrated that GRACE missions show great potential in various fields, e.g., detection of terrestrial water storage [14,15,16] and groundwater storage [17,18]. Swenson et al. [14] derived region-scale water storage by applying averaging kernels to a realistic synthetic GRACE gravity signal within North American river basins. Results indicated that the accuracy might be better than 1 cm for regions with 400,000 km^2^ or larger areas. Rodell et al. [17] simulated groundwater storage based on GRACE and hydrological modeling system, and the results showed that it was being depleted at a mean rate of 4.0 ± 1.0 cm/yr equivalent height of water over the Indian states of Rajasthan, Punjab and Haryana (including Delhi).

Although remarkable achievements have been made in large-scale areas, the application of GRACE observations in local areas is very limited due to the coarse spatial resolution (~200,000 km^2^) [19]. Consequently, some downscaling methods have been proposed for providing high-resolution GRACE products, which are mainly divided into two categories: dynamic downscaling and statistical downscaling [20,21], respectively. Normally, dynamic downscaling tends to achieve regional downscaling by using the initial boundary conditions of global climate models [22,23] directly. For example, Eicker et al. [24] assimilated GRACE-derived TWSA into the WaterGAP Global Hydrology Model by introducing a new Kalman filter method, which can provide reasonable results in the Mississippi river basin. Although data assimilation methods remain consistent in the physical process, some shortcomings still require to be considered [25]. The implementation of data assimilation is relatively complicated [26], and its accuracy is subject to the full error covariance matrix of GRACE observations and hydrological models [27,28].

Compared to dynamic downscaling, statistical downscaling usually establishes the linear or non-linear relationships between input and target variables, aiming to produce local-scale information [29,30]. Initially, linear regression models are employed to downscale GRACE products [31,32,33]. For example, Ning et al. [31] achieved the downscaling of GRACE data in parts of Yunnan by constructing an empirical regression model based on the water balance equation, and the results indicated the feasibility for downscaling GRACE data. Yin et al. [33] proposed a new statistical downscaling algorithm by building the relationship between multi-source evapotranspiration (ET) products and GWSA in the North China Plain, which obtained desirable downscaled results but limited by the strong correlation between TWS and ET. Practically, the relationships between predictor and predictand tend to be non-linear rather than linear. The development of machine learning algorithms provides effective measures to quantify the complicated relationship by constructing non-linear models. Artificial neural networks (ANNs) have the capabilities of simulating complex hydrological characteristics to an arbitrary degree of accuracy [34,35]. This makes ANN becomes an attractive measure in the downscaling researches, which have been applied to some typical regions, e.g., the Northern High Plains [36], California’s Central Valley [34], and the Lower Peninsula of Michigan [26]. Similarly, some tree-based machine learning algorithms (e.g., random forest (RF) and gradient boosting decision tree (GBDT)) become popular in regression tasks with the advantages of simplicity and effectiveness. The RF algorithm has been utilized to downscale GRACE observations and obtained satisfactory results in some areas [37,38]. As a kind of ensemble machine learning algorithm, GBDT performs well in constructing non-linear regression models, which is often employed to forecast ET [39] and urban flood [40,41], but rarely in GWLA. Furthermore, the multi-stage machine learning algorithm may have more powerful expressive performance than a single algorithm in downscaling GRACE products. For example, Seyoum et al. [42] designed a two-layer boosted regression trees (BRT) model by utilizing GRACE data and hydrological variables in a glacial aquifer system of the United States, which can predict groundwater level anomalies (GWLA) with a high spatial resolution. 

The North China Plain (NCP), which is the political, economic, and cultural center of China [43], has been suffering from water shortage and over-exploitation of groundwater for a long period [44,45,46]. Some downscaling researches have been conducted in the area, aiming to provide high-resolution water storage estimates [33,37,47]. Water resources managers are often more concerned with information about water levels at specified locations, while few studies are conducted with respect to this aspect. To overcome this limitation, this study proposes a machine learning-based fusion model, aiming to downscale GRACE-derived TWSA to higher spatial resolution products and predict higher-accuracy groundwater level anomalies (GWLA). The structure of this study is organized as follows. The overview of the NCP and the data sources are introduced in Section 2. Section 3 describes the structure and construction of the machine learning-based fusion model. Section 4 provides the results of the downscaling and prediction module in the NCP. The discussions and conclusions are presented in Section 5 and Section 6, respectively.

## 2. Study Area and Data 

### 2.1. Study Area

**Figure 1:**
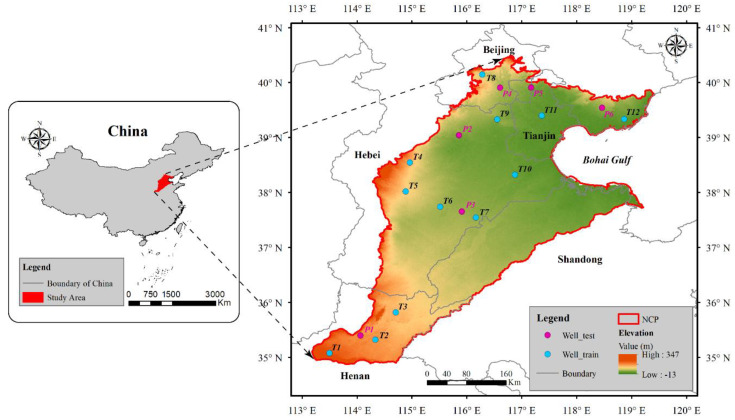
Location and digital elevation model map of the NCP. The blue and magenta dots represent the training and testing wells, respectively.

The North China Plain, located in the eastern coastal region of China, lies between latitude 35° N–41° N and longitude 113° E–120° E (Figure 1). It is one of the three great plains in China, covering an area of approximately 140,000 km^2^ [48]. The NCP is a central agricultural area in China, which produces about one-fourth of the country’s total grain yield [10]. The main crops include winter wheat and summer maize, and the NCP supplies more than 50% of the wheat and approximately 33% of the maize production in China [49]. 

The NCP belongs to a continental monsoon climate with an annual average temperature between 8 and 15 °C [33,50]. The annual precipitation, most of which occurs during the growth period of summer maize, ranges from 500 to 600 mm, and annual evaporation is 900–1400 mm [51,52]. The NCP contains a shallow unconfined aquifer (40–60 m) and three confined aquifers of different depths (120–170 m, 250–360 m, and 400–600 m) [53]. 

### 2.2. Data

**Figure 2:**
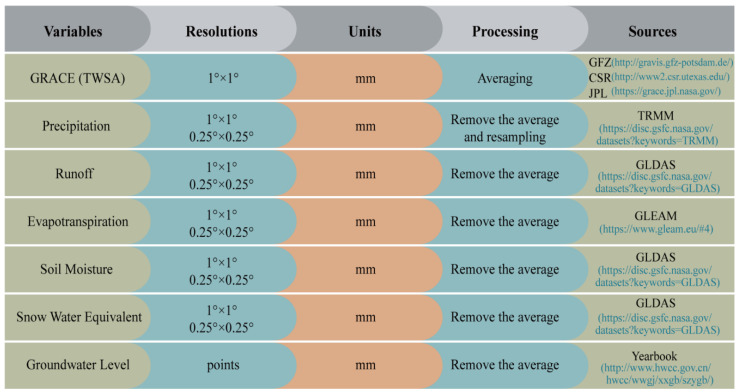
Summary of variable information (resolutions, units, processing, and sources) employed in the fusion model.

The fusion model, proposed in this study, is designed based on the water balance principle and machine learning methods (i.e., multiple linear regression and GBDT). Several datasets (terrestrial water storage anomalies, precipitation, runoff, evapotranspiration, soil moisture, snow water equivalent, and groundwater level) are chosen to owe to their close relationship with groundwater storage changes, as shown in Figure 2.

**Figure 3:**
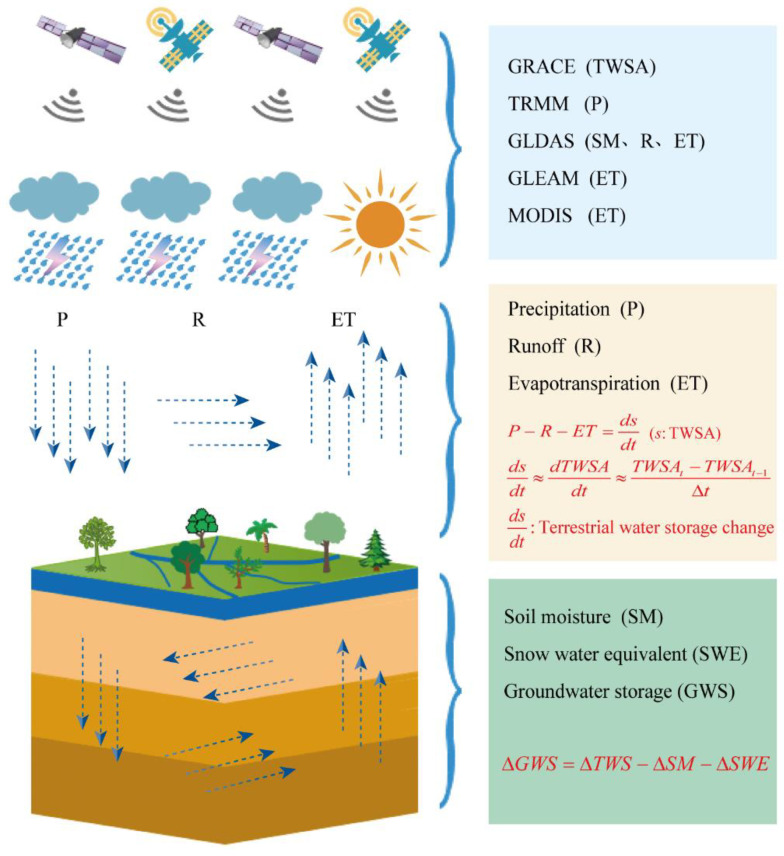
Sketch map of the water cycle.

Specifically, some variables should be resampled from 0.25° to 1° for matching the spatial availability of GRACE products, and the study period covers from January 2005 to December 2014, with a total of 120 months. The schematic diagram of the water balance principle is shown in Figure 3.

#### 2.2.1. GRACE TWSA

The GRACE gravity satellites, jointly developed by NASA (National Aeronautics and Space Administration) and DLR (German Aerospace Center), were launched in 2002 and successfully completed its missions in 2017 [13]. They were designed to track global mass changes or gravity variations using the K-band ranging system and low-low satellite tracking satellite mode [13,54]. In this study, GRACE observations are provided by the Jet Propulsion Laboratory (JPL), Center for Space Research (CSR), and GeoForschungsZentrum Potsdam (GFZ), respectively. The gridded-gain factors are utilized to reduce the leakage error [55], which are available at [56]. Some discrepancies exist among these three solutions due to different processing strategies and tuning parameters [57]. Therefore, we utilize the ensemble average of different solutions as the representative TWS estimates in the following discussion.

#### 2.2.2. TRMM Precipitation

The Tropical Rainfall Measurement Mission (TRMM) is a joint project of NASA and Japan Aerospace Exploration Agency (JAXA), aiming to analyze the impact of rainfall data on weather and climate [58]. The monthly precipitation products (2003–2015) used in this study are the TRMM 3b43 with the spatial resolution of 0.25° × 0.25°. The dataset can be obtained from the Goddard Earth Sciences Data and Information Services Center (GES DISC) [59]. Previous studies [16] have demonstrated that TRMM data match well with gauged stations compared to other remotes sensing products, thus utilized in this study.

#### 2.2.3. GLDAS Data

The Global Land Data Assimilation System (GLDAS) is developed by the Goddard Space Flight Center (GSFC). The primary goal of the GLDAS is to ingest satellite- and ground-based observational data products, using advanced land surface modeling and data assimilation techniques, in order to generate optimal fields of land surface states and fluxes [60]. Up to now, there have been four land surface models (LSM), namely Mosaic [61], Community Land Model (CLM) [62], Noah [63], and Variable Infiltration Capacity (VIC) [64], respectively. The Noah model is selected to provide some water-budget variables, including runoff (R), soil moisture (SM), and snow water equivalent (SWE). The runoff includes surface runoff and underground runoff, and soil moisture is the sum of four soil water layers. The datasets from the Noah model include two kinds of temporal resolutions (3-h and monthly scale) and spatial resolutions (0.25° × 0.25° and 1° × 1°). Monthly datasets are chosen in this study, with the resolutions of 0.25° and 1°, which are available at [65]. 

#### 2.2.4. GLEAM Product

GLEAM (Global Land Evaporation Amsterdam Model) is a set of algorithms dedicated to estimating global evapotranspiration by combining satellite observations and the Priestley and Taylor equation [66]. It has been continuously revised and updated since 2011, and the third version of the model was released in 2017 [67]. The latest version GLEAM v3.3 contains two kinds of data sets (v3.3a and v3.3b), differing in their forcing and temporal coverage [68]. In this study, the GLEAM v3.3a is employed to provide monthly estimates in evapotranspiration, spanning the 36 years from 1980 to 2018. 

#### 2.2.5. Groundwater Level

Monthly groundwater level, collected from the Haihe River Basin Water Resources Bulletin [69], is used to validate the accuracy of the downscaled TWSA and predicted water level. Groundwater monitoring wells are unevenly distributed across the NCP, and there are abnormal jumps, data gaps, and outliers in some wells. Therefore, the collected data require pre-processing as follows: (1) ignore the wells with more missing months and obvious errors; (2) aggregate the groundwater level to pixels values on 0.25° cells by using the simple average of groundwater observations within the pixel; (3) remove the mean value to obtain the groundwater level anomalies data. A total of 18 wells are selected for this study (Figure 1), and 12 wells are used to train the downscaled model, while the remaining 6 wells are used to test the performance of models.

## 3. Methods

### 3.1. Gradient Boosting Decision Tree

The gradient boosting decision tree is an algorithm that combines a series of weak learners into one strong learner [70]. Although the GBDT algorithm can be used for both classification and regression tasks, we only consider the latter in this study. Gradient boosting of regression trees can produce competitive, highly robust, interpretable procedures for all models, especially appropriate for mining less than clean data [70]. Different from other traditional regression methods, the GBDT algorithm obtains the global convergence by following the direction of the negative gradient, which will decrease the running time for getting the results [71]. The calculation core of GBDT is to learn and forecast by continually passing the residual sum of the conclusions of all the previous decision trees until the sum of the predicted values, and the input target residuals are minimized. Consequently, this study tries to develop the downscaling and prediction models based on the regression function of the ensemble algorithm.

### 3.2. Downscaling Approach Based on the Noah Model

The GRACE-derived TWSA includes plenty of water storage information (e.g., groundwater storage, soil moisture, and snow water equivalent), and part of these variables can also be simulated by the GLDAS-Noah model. Consequently, we can obtain two kinds of TWSA products from GRACE missions and the Noah model, respectively. Some discrepancies will exist in these products due to the absence of groundwater storage and anthropogenic factor in the Noah model. In order to obtain finer-resolution and higher-accuracy TWSA products, a regression model (called GRACE-Noah model for short) is employed to downscale GRACE data in this study, which can incorporate GRACE products and the Noah model [32,72]. In the GRACE-Noah model, the simulated TWSA is treated as “truth”, and the bias can be calculated by the following formula:

(1) $$B=TWSA_{Noah,i}^{1}-TWSA_{GRACE,i}^{1},$$

where *B* is the bias of two kinds of TWSA products on the 1° grid; $TWSA_{Noah,i}^{1}\;$is the normalized TWSA simulated by the Noah model; $TWSA_{GRACE,i}^{1}\;$is the normalized TWSA derived from GRACE. The subscript 1 represents the 1 degree. Then, the GRACE products can be downscaled from 1° to 0.25° by the formula:

(2) $$TWSA_{end,i}^{0.25}=TWSA_{Noah,i}^{0.25}-\frac{B\times A\times TWSA_{Noah\_pre,i}^{0.25}}{\sum \left(TWSA_{Noah\_pre,i}^{0.25}\times a_{i}\right)},$$

where $TWSA_{end,i}^{0.25}$ is the downscaled GRACE TWSA with the 0.25° spatial resolution, $TWSA_{Noah,i}^{0.25}$ is the normalized TWSA simulated form the Noah model, *B* is the bias obtained by Formula (1), *A* is the area of the 1° grid (m^2^), $TWSA_{Noah\_pre,i}^{0.25}$ is the pre-normalized TWSA simulated from the Noah model, $a_{i}$ is the area of the 0.25° grid (m^2^).

### 3.3. Multiple Linear Regression

Multiple Linear Regression (MLR) is a regression modeling method with multiple independent and dependent variables [73,74]. The essential parameter estimation method is the least squares method, which is used mainly to find the best function by minimizing the sum of squares of errors. Compared to the single regression model, the multiple linear regression model is more practical and accurate in simulating the relationship between independent and dependent variables, which can better achieve the prediction and estimation of TWSA products. The following formula can describe the MLR method used in this study:

(3) $$y=a_{0}+a_{1}x_{1}+a_{2}x_{2}+\cdots +a_{n}x_{n},$$

where y is the dependent variable (TWSA), $x_{1},x_{2},\cdots ,x_{n}$ represent the independent variables (e.g., P, R, and ET), $a_{0}$ is the constant value, $a_{1},a_{2},\cdots ,a_{n}$ are the weights of *n* variables.

### 3.4. Fusion Model Design

**Figure 4:**
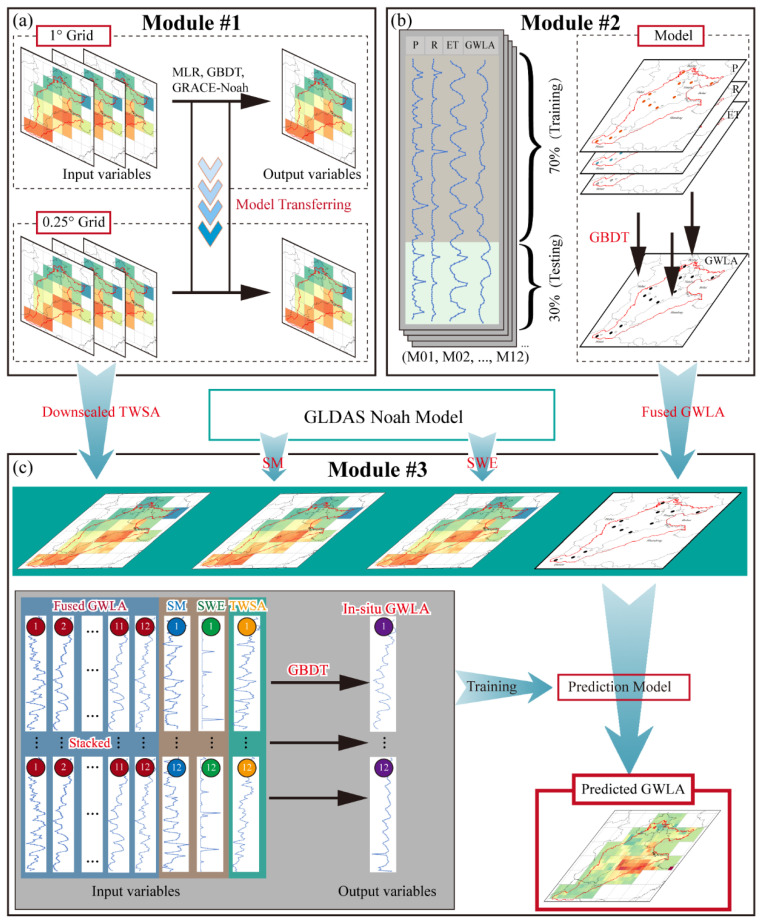
The conceptual map of the machine learning-based fusion model. (**a**) Downscaling module; (**b**) Data fusion module; (**c**) Prediction module.

To obtain high-resolution and high-accuracy GWLA, the machine learning-based fusion model is developed within the NCP, which mainly consists of three modules. Specifically, Module #1 is used to downscale GRACE-derived TWSA from 1° to 0.25° by using different algorithms (MLR, GBDT, and GRACE-Noah), which is also called the downscaling module. Module #2 is employed to incorporate climate variables with in-situ levels based on the GBDT algorithm, which is also named as the data fusion module. With respect to Module #3, it accepts the downscaled TWSA from Module #1 and the fused GWLA from Module #2. Then, these variables are integrated into a big model for obtaining GWLA in the whole study area, and as module is named the prediction module. The conceptual map of the fusion model is shown in Figure 4.

#### 3.4.1. Module #1 for Downscaling

This study utilizes two traditional machine learning models (MLR and GBDT) and one hydrological model (GLDAS-Noah) to downscale GRACE-derived TWSA into the higher resolution, as shown in Figure 4a. The detailed process of this module is described as follows:

*Step 1*: Climate variables (P, R, and ET) are chosen as the predictor based on the water balance equation, and GRACE-derived TWSA is selected as the predictand. The climate variables are resampled from 0.25° to 1° in accordance with that of GRACE products.

*Step 2*: Under the coarse resolution, these downscaling models are developed for each 1° grid based on the input variables (P, R, and ET) and the output variable (TWSA). Each model is continuously trained and tested by adjusting the core parameters until it can achieve satisfactory metrics (e.g., root mean square error (RMSE) [75], Nash-Sutcliffe efficiency coefficient (NSE) [76,77], and correlation coefficient (CC) [78]), and the mean absolute error (MAE) [79].

*Step 3*: The relationship is constructed between predictors and predictands at the resolution of 1°. It is assumed that the relationship is still accurate under different spatial resolutions. The downscaled TWSA can be obtained in the study area by employing higher-resolution variables into these models.

*Step 4*: The bias of simulated TWSA and GRACE-derived TWSA is calculated at the resolution of 1° for each grid. Then, these values are resampled to 0.25 based on the Kriging interpolation [80] and assigned to the corresponding grid.

*Step 5:* GWSA can be isolated from the downscaled TWSA based on the auxiliary information provided by the Noah model. Then, the downscaling performance of the machine learning (MLR and GBDT) can be validate by comparing the downscaled GWSA with the groundwater measurements.

#### 3.4.2. Module #2 for Data Fusion

The data fusion model is proposed to incorporate some climate variables (P, R, and ET) information with the in-situ well measurements, which are utilized to control other grids in the Module #3. Monthly groundwater level data of 18 observation wells are collected from the Haihe River Basin Water Resources Bulletin. In this module, we select 12 wells as the control wells based on the distribution of shadow groundwater wells. As for each well, the corresponding shuffled dataset, used in the data fusion model, is divided into two parts for training (70%) and testing (30%). At last, we determine 12 most reasonable models by continuously adjusting the structure of the GBDT model based on these metrics (e.g., RMSE, NSE, and CC). The model design flowchart is shown in Figure 4b (Module #2).

#### 3.4.3. Module #3 for Prediction

To get high-quality GWLA products, the prediction module is developed by using the results from the first two modules and other climate variables (e.g., SM, SWE, and in-situ measurements), as shown in Figure 4c (Module #3). In the module, 12 observation wells are selected as the training wells, and the remaining 6 wells are used to evaluate the performance of the prediction model. In order to construct the prediction model, we select 15 characteristics as the predictors, including four kinds of variables. As for each training well, we construct a sub-dataset, which includes 12 fixed variables (12 fused GWLA) and 3 changed water-budget variables (SM, SWE, and TWSA). Then, 12 sub-datasets are stacked into a training matrix with the dimensions of 1440 × 15. By continuous adjusting and training, an ideal prediction model is developed in the NCP. It assumes that the model is applicable within all study areas; we can obtain all GWLA by constructing and employing sub-datasets into the model for each grid.

### 3.5. Model Evaluation and Data Analysis Standards

In order to evaluate the performance of the fusion model, four indices are used as the evaluation criteria, namely, RMSE, MAE, NSE and CC. The specific expressions are as follows:

(4) $$\mathrm{RMSE}=\sqrt{\frac{1}{n}\sum_{i=1}^{n}{\left(Y_{i}-X_{i}\right)}^{2}},$$

(5) $$\mathrm{MAE}=\frac{1}{n}\sum_{i=1}^{n}\left|Y_{i}-X_{i}\right|,$$

(6) $$\mathrm{NSE}=1-\frac{\sum_{i=1}^{n}{\left(Y_{i}-X_{i}\right)}^{2}}{\sum_{i=1}^{n}{\left(X_{i}-\bar{X}\right)}^{2}},$$

(7) $$\mathrm{CC}=\frac{\sum_{i=1}^{n}\left(X_{i}-\bar{X}\right)\left(Y_{i}-\bar{Y}\right)}{\sqrt{\sum_{i=1}^{n}{\left(X_{i}-\bar{X}\right)}^{2}}\sqrt{\sum_{i=1}^{n}{\left(Y_{i}-\bar{Y}\right)}^{2}}},$$

where $X_{i}$ and $Y_{i}$ represent two independent datasets with the mean values of $\bar{X}\;$and $\bar{Y}$, $X_{i}$ represents the simulated value, $Y_{i}$ represents the measured value, n means the total number of samples. As for the RMSE and MAE, the smaller the values are, the higher the accuracy of the model. Similarly, the closer the values of CC and NSE are to 1, the more consistent the simulated and measured values are.

## 4. Results

### 4.1. Evaluation of Downscaling Models

The performance of three downscaling models is evaluated from two perspectives, which are spatial and temporal resolution. The detailed descriptions and results are as follows:

#### 4.1.1. Spatial Resolution

**Figure 5:**
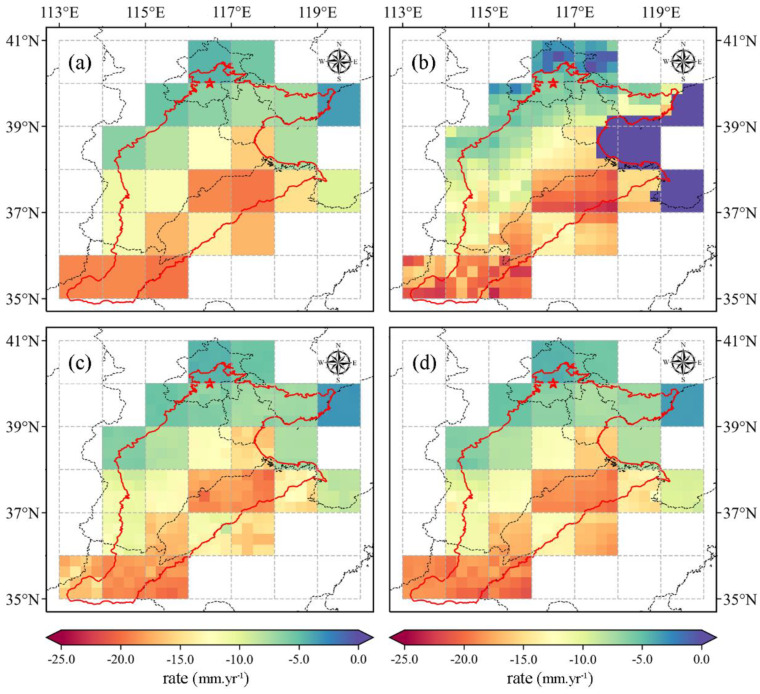
Spatial distribution of TWSA trends from 2005 to 2015. (**a**) GRACE-derived TWSA, (**b**) downscaled results based on GRACE-Noah, (**c**) downscaled results based on GBDT, and (**d**) downscaled results based on MLR.

Figure 5 shows the long-term trends of GRACE-derived TWSA and three downscaled results in the NCP. It can be found that the spatial distribution characteristics of TWSA are basically consistent with that of downscaled results. In general, the downscaled results can capture the sub-grid heterogeneity, while preserving the TWSA characteristic at the original scale. An obvious downtrend is observed in the southwestern region of the NCP, which is located in the conjunction area of Hebei and Henan province. This is mainly caused by intensive agricultural activities, which is in accordance with previous studies [16,81]. Furthermore, the downtrend becomes serious from the northern to the southern parts, with the trend of −4.96 mm/yr and −18.87 mm/yr, respectively. Additionally, there are some outlier values near the Bohai region in the GRACE-Noah model. The possible reason is that there are large uncertainties in the forcing data of the GLDAS-Noah model.

**Figure 6:**
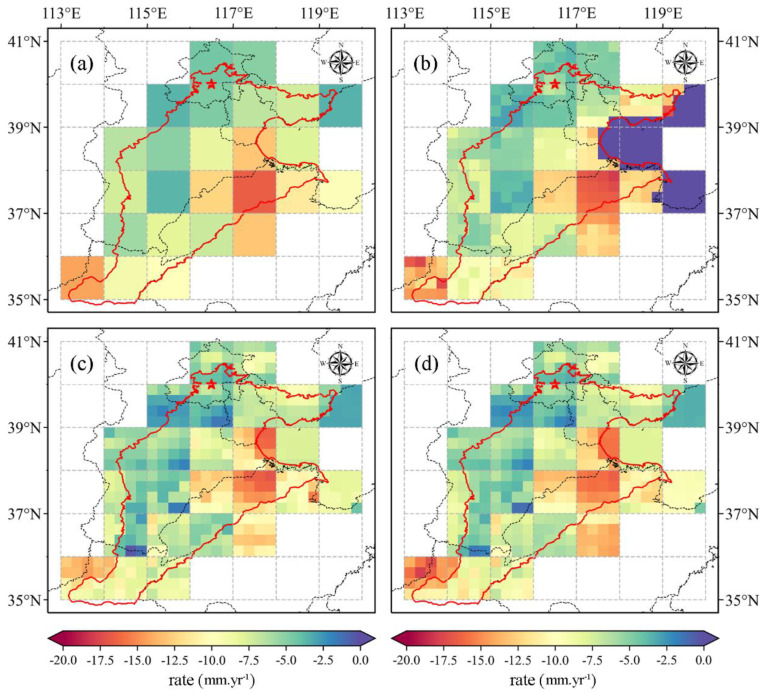
Spatial distribution of the GWSA trend from 2005 to 2015. (**a**) GRACE -derived TWSA, (**b**) downscaled results based on GRACE-Noah, (**c**) downscaled results based on GBDT, and (**d**) downscaled results based on MLR.

Additionally, the long-term trends of GRACE-based and downscaled GWSA are shown in Figure 6 during the period from 2005 to 2015. The larger decreasing trend is also detected in the Southern regions, which is consistent with that of TWSA. It is worth noting that the range of trends varies from −16.61 mm/yr to −1.41 mm/yr, which is only a bit smaller than that of TWSA (from −29.70 mm/yr to −2.08 mm/yr), indicating that the slope of TWSA is mainly caused by GWSA estimates. What is more, we can find that the East Central Plain is the most serious region, and it may be due to the over-exploitati6on of deep groundwater storage [82].

#### 4.1.2. Temporal Resolution

**Figure 7:**
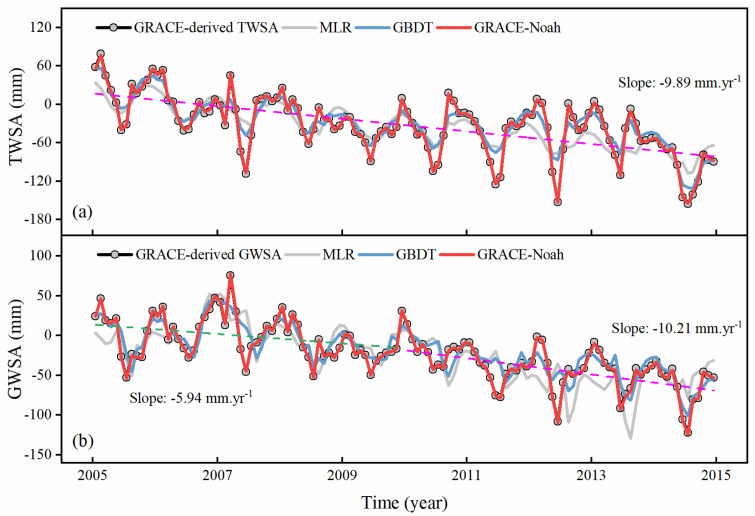
Time-series trends of (**a**) TWSA and (**b**) GWSA before and after downscaling in the whole NCP. The black line shows the time-series trend of GRACE-derived products, while grey, blue, and red lines repBresent downscaled results (MLR, GBDT, and GRACE-Noah).

In order to evaluate the downscaled results more intuitively, the time series of TWSA and GWSA are plotted in Figure 7. Similar decreasing trends can be observed in TWSA and GWSA from 2005 to 2015, with the trend of −9.89 mm/yr and −8.45 mm/yr, respectively. With respect to GWSA, the downtrend intensifies with the slope increasing from −5.94 mm/yr to −10.21 mm/yr. The downscaled water storage estimates based on the GLDAS-Noah model are well correlated with the results at the original resolution, with the correlation up to 0.99, and the acceptable RMSE value of 1.49 mm (Table 1). The worse performance can be found in the MLR model, and the possible reason is that there is some information missing during the process of downscaling. Based on these above discussions, we choose the downscaled results of GRACE-Noah as representative values, thus used in the following discussions.

### 4.2. Results of Data Fusion

Based on previous studies, the GBDT algorithm is employed to construct the regression model with the advantages of robustness, efficiency, and simplicity [42,70]. Specifically, the GBDT model is designed for 12 in-situ wells, which are selected based on their spatial distribution and data quality. Each model is developed for incorporating some climate variables (P, R, and ET) into in-situ observations.

**Figure 8:**
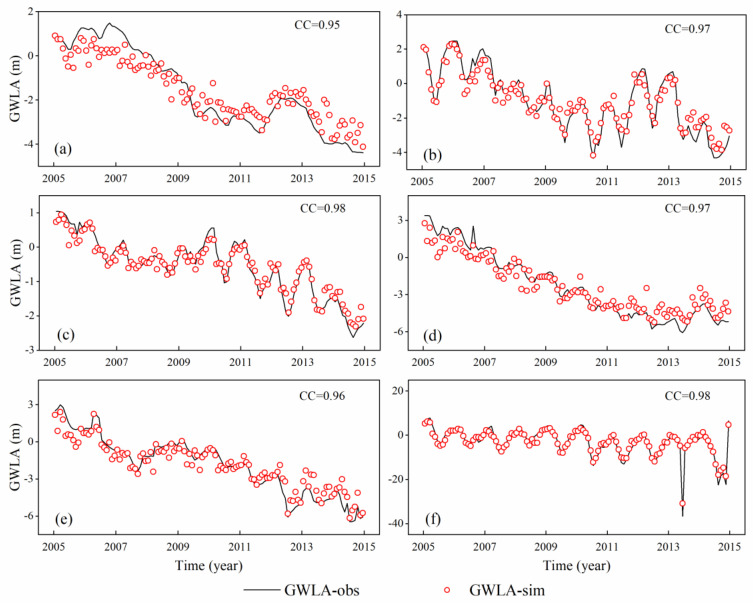
Time-series comparison of in-situ and simulated GWLA data. (**a**–**f**) represent the corresponding wells (T1–T6).

The performance of models and time-series comparison results before and after fusion are shown in Table 2 and Figure 8, respectively. It can be found in Table 2 that 12 data fusion models show good performances with the average RMSE, MAE, NSE, and CC values of 1.10 m, 0.87 m, 0.91, and 0.97, respectively. 

Six monitoring wells are utilized to evaluate the applicability of the fusion model from the time series trend, and the verification results indicate that all of them perform ideal CC values (0.95, 0.97, 0.98, 0.97, 0.96, and 0.98). Then, the fused GWLA data of 12 wells are regarded as the control wells and used as the input variables of the prediction model in the Module #3.

### 4.3. Prediction Performance Analysis

**Figure 9:**
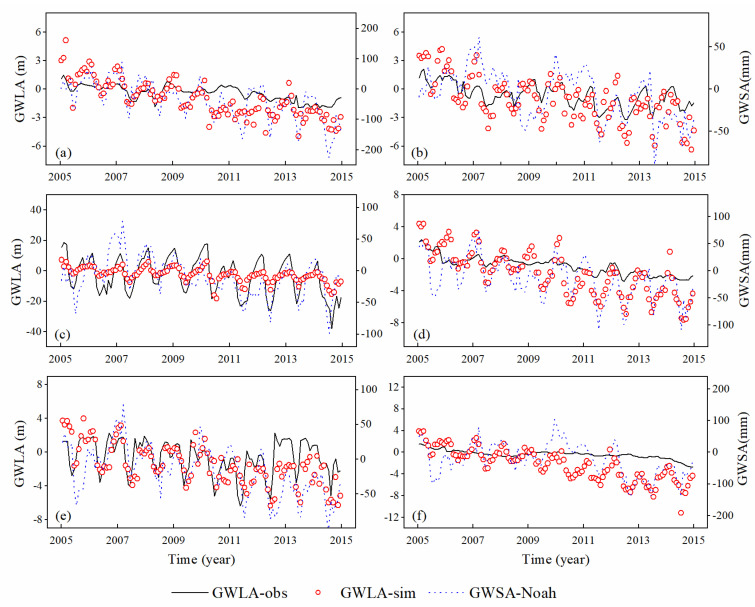
Comparison of test wells data before and after the prediction model. (**a**–**f**) represent the corresponding wells (P1-P6). GWLA-obs, GWLA-sim, and GWSA-Noah represent the in-situ GWLA, simulated GWLA, and GRACE-derived GWSA, respectively.

Based on the downscaled TWSA and 12 control wells, the prediction model is developed to forecast the groundwater level at the 0.25° pixel scale. The remaining six wells are utilized to evaluate the accuracy of the prediction model, as shown in Figure 9. Results reveal that the predicted GWLA is reasonable in the first five wells but worse in the last one. This is attributed to the fact that the P6 well is close to the Bohai region with poor quality of in-situ measurements. On the whole, there will be a certain deviation between the predicted value and the in-situ value of all wells, but the overall trend is basically the same.

### 4.4. Verification of In-Situ Observations

**Figure 10:**
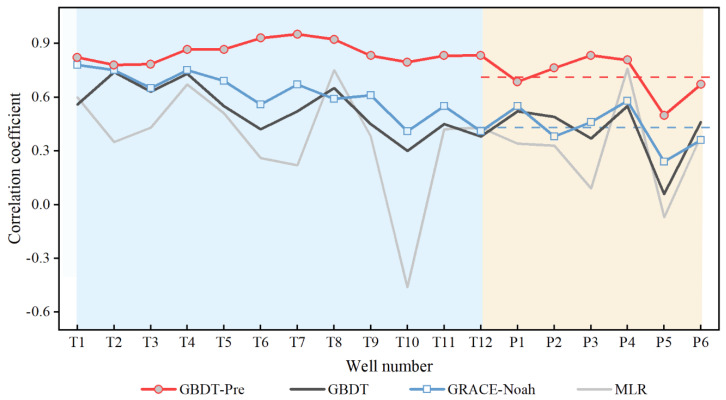
Correlation coefficient comparison before and after prediction. The blue dashed line and the red dashed line show the mean CC values of all wells before and after modeling, respectively.

To further explore the applicability of the machine learning-based fusion model, 18 monitoring wells are collected to evaluate the simulated results, and the comparison is shown in Table 3 and Figure 10. The verification includes two parts, which are the verification of downscaled results and the verification of predicted results.

As for downscaled results, all of 18 total wells are utilized to evaluate the performance of models, and three downscaling models present reasonable results with the mean CC values of 0.36 (MLR), 0.49 (GBDT), and 0.56 (GRACE-Noah), respectively. Although the MLR and GRR models may show better performance in several wells, such as P2, P4, and P6 wells, the values are close to the GRACE-Noah model. Moreover, other wells show obviously better performances in the GRACE-Noah model, especially in the T10 well with the CC values of −0.46 (MLR), 0.30 (GBDT), and 0.41 (GRACE-Noah). Consequently, the GRACE-Noah model is considered to be the optimum downscaling model in this study, followed by the GBDT model, which is consistent with the result Section 4.1. Therefore, the groundwater estimates based on the GRACE-Noah model are used to compare with the predicted products.

As for the predicted results, the CC values before and after prediction are compared in Figure 10. The light blue areas on the left represent the results of 12 control wells, while the light yellow areas stand for the results of 6 predicted wells. It can be seen that the mean CC between GRACE-derived GWSA (GRACE-Noah) and observed GWLA is 0.43, while the predicted results increase to 0.71 against downscaled values. Moreover, all of the CC values between the predicted results and in-situ GWLA are better than the downscaled results, especially in the P6 well, whose performance is higher than the expected result with the CC value of 0.67 (Figure 9f and Figure 10). Overall, the prediction model presents an excellent performance in simulating the changing trend but may be insufficient in numerical prediction.

## 5. Discussion

### 5.1. Efficacy of the Fusion Model

Performance metrics from the downscaled and the predicted modules indicate that the machine learning-based fusion model can successfully achieve the purpose of downscaling GRACE-derived TWSA and predicting GWLA. In the Module #1, three kinds of methods are employed to downscale GRACE observations into 0.25°, which are GBDT, MLR, and GRACE-Noah, respectively. Results indicate that the GRACE-Noah model outperforms the other two models, especially in terms of temporal scales (Figure 7). The possible reason may be that the downscaled algorithm based on the Noah model can effectively assign the discrepancies between GRACE and hydrological model into the pixel at higher resolution, thereby preserving the integrity of climate information at the coarse resolution. With respect to the Module #2, it incorporates the information of variables (P, R, and ET) into GWLA based on the 12 models, which are built for each control well using the GBDT machine learning method. As shown in Table 3, each model reveals excellent performance with the CC values ranging from 0.95 to 0.98. In the Module #3, the downscaled TWSA from the Module #1 and fused GWLA from the Module #2 are taken into account in the prediction model. It can be seen in Figure 10 that the prediction model performs reasonably in simulating dynamic changes in GWLA, with the CC values ranging from 0.50 to 0.95. In general, the fusion model developed in this study present satisfactory performance in downscaling and prediction phases within the NCP. 

### 5.2. Limitations and Outlook

Based on these above discussion, the developed fusion model can effectively downscale GRACE observations and predict high-quality GWLA at pixel scales. However, the highest resolution of predicted results is mainly determined by the resolution of climate variables (Module #1) and water storage estimates (Module #3). The water balance variables are the most widely used in previous studies [31,37], provided at the maximum resolution of 0.25°. Similarly, the water storage components are obtained from the GLDAS-Noah model, which provides simulated outputs at the resolutions of 1° and 0.25°. Consequently, the target resolution of downscaled is 0.25° in this study. 

Although the fusion method performs promise in this study, we will make improvements from the following aspects in the future. On the one hand, only three kinds of climate variables are taken into account in the downscaling module. Theoretically, more input variables have the potential to improve the accuracy of downscaling results, such as temperature and normalized difference vegetation index. What is more, this study only selects 12 groundwater levels as the control well, restricted by the limited in-situ measurements. If more observed data are obtained, the fusion model may perform better than it does now. Of course, the performance of the fusion model is also limited the actual situation of different study areas.

## 6. Conclusions

Based on TWSA products derived from GRACE gravity satellites, fruitful results in research on groundwater levels have been achieved in large-scale areas. However, due to the coarse spatial resolution of GRACE observations, the ability to study the changes in groundwater levels is limited in small-scale areas. Consequently, this study conduct meaningful research on downscaling GRACE-derived TWSA and predicting high-quality GWLA based on the machine learning algorithms, and the results are summarized as follows:

(1) The machine learning-based fusion model, including three modules (downscaling module, data fusion module, and prediction module), is proposed in the NCP based on the empirical relationships between GRACE and climate drivers. These modules are both independent and integrated because the first two modules provide input variables for the prediction module while exhibiting their functions.
(2) GRACE-derived TWSA is downscaled from 1° to 0.25° by utilizing three downscaling models (MLR, GBDT, and GRACE-Noah models). From the spatial resolution and temporal resolution, we compare the performances of downscaling models, and the findings indicate that the GRACE-Noah model performs the best performance, with the CC value of 0.99 and RMSE value of 1.49 mm in the whole study area. What is more, the verification results with in-situ observations of 18 wells also indicate the same result, with acceptable CC values ranging from 0.24 to 0.78.
(3) Based on the downscaled and fused results, the prediction model is developed to obtain the GWLA within the whole NCP, and the verification results (CC values ranging from 0.50 to 0.95) indicate that the performance in simulating the long-term trend is ideal but may be insufficient in numerical prediction. Further, the average CC values of 6 test wells are calculated after prediction, which performs that the predicted result (0.71) is 65.12% higher than the downscaled result (0.43).

Overall, the proposed fusion model can effectively implement the downscaling of GRACE products and the prediction of high-accuracy GWLA in the NCP. To some extent, the fusion model can provide some suggestions to obtain and understand the dynamics of water resources for some areas with no or less in-situ measurements. However, the output spatial resolution and accuracy of the fusion model are limited by the climate variables and the water storage components. If higher-resolution and higher-precision climate variables can be obtained in the future, the fusion model may have the potential to obtain higher quality products (TWSA and GWLA).

## Figures and Tables

**Table 1:** Performance of downscaling models (RMSE, MAE, NSE, and CC).

	Models	RMSE (mm)	MAE (mm)	NSE	CC
TWSA	GRACE-Noah	1.49	1.17	0.99	0.99
	GBDT	18.00	10.20	0.85	0.93
	MLR	28.32	16.84	0.67	0.79
GWSA	GRACE-Noah	1.24	0.81	0.99	0.99
	GBDT	17.08	9.78	0.75	0.87
	MLR	27.23	15.81	0.36	0.68

**Table 2:** Performances of 12 GBDT models (RMSE, MAE, NSE, and CC).

Model	Grid	RMSE (m)	MAE (m)	NSE	CC
M01	T1	0.72	0.59	0.85	0.95
M02	T2	0.55	0.44	0.91	0.97
M03	T3	0.23	0.18	0.93	0.98
M04	T4	0.85	0.68	0.90	0.97
M05	T5	0.74	0.57	0.90	0.96
M06	T6	1.58	1.20	0.94	0.98
M07	T7	3.03	2.38	0.94	0.98
M08	T8	1.40	1.16	0.87	0.96
M09	T9	0.61	0.47	0.91	0.97
M10	T10	1.26	1.05	0.95	0.97
M11	T11	0.72	0.58	0.88	0.96
M12	T12	1.51	1.19	0.88	0.96
Mean		1.10	0.87	0.91	0.97

**Table 3:** Performances of the machine learning-based fusion model (CC).

	Downscaled Results			Predicted Results
Wells	MLR	GBDT	GRACE-Noah	GBDT-Pre
T1	0.60	0.56	0.78	0.82
T2	0.35	0.74	0.75	0.78
T3	0.43	0.63	0.65	0.78
T4	0.67	0.73	0.75	0.87
T5	0.51	0.55	0.69	0.87
T6	0.26	0.42	0.56	0.93
T7	0.22	0.52	0.67	0.95
T8	0.75	0.65	0.59	0.92
T9	0.38	0.45	0.61	0.83
T10	−0.46	0.30	0.41	0.79
T11	0.42	0.45	0.55	0.83
T12	0.43	0.38	0.41	0.83
P1	0.34	0.52	0.55	0.69
P2	0.33	0.49	0.38	0.76
P3	0.09	0.37	0.46	0.83
P4	0.76	0.55	0.58	0.81
P5	−0.07	0.06	0.24	0.50
P6	0.38	0.46	0.36	0.67
Mean	0.36	0.49	0.56	0.80

## Data Availability

No new data were created or analyzed in this study. Data sharing is not applicable to this article.
